# Regulatory and functional divergence among members of *Ibβfruct2*, a sweet potato vacuolar invertase gene controlling starch and glucose content

**DOI:** 10.3389/fpls.2023.1192417

**Published:** 2023-06-27

**Authors:** Kai Zhang, Zhengdan Wu, Xuli Wu, Haohao Han, Xisan Ju, Yonghai Fan, Chaobin Yang, Daobin Tang, Qinghe Cao, Jichun Wang, Changwen Lv

**Affiliations:** ^1^ College of Agronomy and Biotechnology, Southwest University, Beibei, Chongqing, China; ^2^ Key Laboratory of Biology and Genetic Breeding for Tuber and Root Crops in Chongqing, Beibei, Chongqing, China; ^3^ Engineering Research Center of South Upland Agriculture, Ministry of Education, Chongqing, China; ^4^ Xuzhou Institute of Agricultural Sciences in Jiangsu Xuhuai District/Sweet potato Research Institute, Chinese Academy of Agricultural Sciences, Xuzhou, China

**Keywords:** sweet potato, invertase, duplicated gene, sequence variation, promoter activity

## Abstract

Sweet potato [*Ipomoea batatas* (L.) Lam.] is an important food and industrial crop. Its storage root is rich in starch, which is present in the form of granules and represents the principal storage carbohydrate in plants. Starch content is an important trait of sweet potato controlling the quality and yield of industrial products. Vacuolar invertase encoding gene *Ibβfruct2* was supposed to be a key regulator of starch content in sweet potato, but its function and regulation were unclear. In this study, three *Ibβfruct2* gene members were detected. Their promoters displayed differences in sequence, activity, and *cis*-regulatory elements and might interact with different transcription factors, indicating that the three *Ibβfruct2* family members are governed by different regulatory mechanisms at the transcription level. Among them, we found that only *Ibβfruct2-1* show a high expression level and promoter activity, and encodes a protein with invertase activity, and the conserved domains and three conserved motifs NDPNG, RDP, and WEC are critical to this activity. Only two and six amino acid residue variations were detected in sequences of proteins encoded by *Ibβfruct2-2* and *Ibβfruct2-3*, respectively, compared with *Ibβfruct2-1*; although not within key motifs, these variations affected protein structure and affinities for the catalytic substrate, resulting in functional deficiency and low activity. Heterologous expression of *Ibβfruct2-1* in Arabidopsis decreased starch content but increased glucose content in leaves, indicating *Ibβfruct2-1* was a negative regulator of starch content. These findings represent an important advance in understanding the regulatory and functional divergence among duplicated genes in sweet potato, and provide critical information for functional studies and utilization of these genes in genetic improvement.

## 1 Introduction

Sweet potato [*Ipomoea batatas* (L.) Lam.] is grown widely throughout the world due to its high yield potential, low input requirement, and adaptability under a range of environmental conditions (Zhou et al., 2015; Dong et al., 2019). It yields a large amount of energy per unit area per unit of time (Nedunchezhiyan et al., 2012). The storage root of sweet potato is rich in starch, which is the principal storage carbohydrate in plants. Starch levels in the storage root are 20% to 30% of the wet weight (Srichuwong et al., 2012) and 50% to 80% of the dry weight (Zhou et al., 2015). The high starch content and reliable starch yield of sweet potato render it an excellent raw material for starch-based industries and environmentally friendly ethanol biofuel production (Zhou et al., 2015; Lyu et al., 2021). Indeed, sweet potato may have an even greater potential than maize (*Zea mays*) as an ethanol source (Nedunchezhiyan et al., 2012; Srichuwong et al., 2012; Koçar and Civas, 2013). The starch content of storage roots is an important trait of sweet potato, which influence post-harvest processing, affecting energy consumption and CO_2_ emission in ethanol biofuel production and thereby controlling the quality and yield of industrial products and ethanol.

Sweet potato is a highly heterozygous hexaploid. Its genome is large and complex, consisting of a large number of small chromosomes (2*n* = 6*x* = 90), which complicates genetic studies of sweet potato (Yan et al., 2022). Recent studies have shown that sweet potato originated from the hybridization of a diploid and a tetraploid progenitor and comprises two B_1_ and four B_2_ component genomes (B_1_B_1_B_2_B_2_B_2_B_2_) (Yang et al., 2017), meaning it contains three very closely related but partly differentiated diploid subgenomes (Gao et al., 2020). However, the polyploid origin, degree of homology, and genomic components of sweet potato remain elusive (Yang et al., 2017; Gao et al., 2020).

During sweet potato evolution, two whole-genome duplication (WGD) events are estimated to have occurred (Yang et al., 2017). WGDs have played an important role in the diversification and adaptive evolution of many polyploid crops (Li et al., 2011; Kan et al., 2022). In these complex, heterologous genomes, numerous duplicated genes have evolved from polyploidization and frequently exhibit expression bias and functional divergence resulting from neofunctionalization and subfunctionalization (Liu et al., 2018; Birchler and Yang, 2022). The number of duplicated genes generated from one progenitor in hexaploid sweet potato, and whether or not these gene family members show divergence in expression and function, remain insufficiently understood. This seriously hampers the genetic improvement of sweet potato through manipulation of key genes controlling important traits.

Vacuolar invertase (VIN) is a vacuole-located invertase (β-fructofuranosidase; EC 3.2.1.26) that irreversibly hydrolyzes sucrose to fructose and glucose and is therefore required for starch and sucrose metabolism and the development of many sink tissues (Ruan et al., 2010; Wang et al., 2010). VIN plays essential roles in osmoregulation and cell expansion, in the regulation of the sugar composition of fruits and storage organs, and in responses to various stresses in plants (Ruan et al., 2010; Tauzin et al., 2014; Wang and Ruan, 2016; Morey et al., 2018). For example, rice (*Oryza sativa*) OsINV2 regulates sugar composition, transport, and grain size (Xu et al., 2019), and white cotton (*Gossypium hirsutum*) GhVIN1 regulates floral organ development, female fertility, and fiber elongation (Wang et al., 2010; Wang and Ruan, 2016). Potato (*Solanum tuberosum*) acid VIN is a critical regulator of cold-induced sweetening (Wiberley-Bradford and Bethke, 2018). Plant acid invertases share several conserved regions, including the three major motifs NDPNG, RDP, and EC. The conserved residues in or around these motifs are crucial for the activity of VIN (Chen et al., 2009; Tauzin et al., 2014). Regulation of acid invertases occurs at the transcriptional and translational levels following various stimuli or environmental changes (Tauzin et al., 2014). In addition, their activity can be regulated at the post-translational level by N-glycosylation and proteinaceous inhibitors.

Few studies on VIN have been reported in sweet potato. Acid invertase (including VIN and cell wall invertase) activity can be induced by wounding treatment (Matsushita and Uritani, 1974). VIN activity increases after storage root formation, continues to intensify, and remains at high levels during storage root bulking; this activity is much higher than that of neutral invertase and insoluble acid invertase and plays important roles in regulating sucrose unloading in storage roots (Liu et al., 2019). *Ibβfruct2* is an important VIN-encoding gene in sweet potato and is expressed in sprouting shoots, immature leaves, stems, and storage roots (Wang et al., 2005). Its expression is suppressed in lines overexpressing the Dof zing finger transcriptional factor *SRF1*, which contains higher levels of starch and lower glucose and fructose content in storage roots than wild-type sweet potato (Tanaka et al., 2009). Expression levels of *Ibβfruct2* vary across developmental stages, and different genotypes possess varied starch contents. Significant correlation between *Ibβfruct2* expression and starch content in storage roots suggests that *Ibβfruct2* might be a key regulator of starch metabolism (Zhang et al., 2017). However, its function and regulatory mechanism in sweet potato remain unclear.

Only one representative *Ibβfruct2* cDNA has been reported; however, a number of *Ibβfruct2* genes with sequence variations have been found in different sweet potato genotypes. Hence, we cloned these genes and analyzed their evolutionary relationships, expression pattern divergence, promoter characteristics, and protein function to comprehensively understand the regulatory and functional divergence among these *Ibβfruct2* family members. The role of *Ibβfruct2* in starch content was also explored. Our results provide critical information for further revealing the roles of key genes in the regulation of important traits and lay a foundation for their utilization in crop improvement through precise genome manipulation.

## 2 Materials and methods

### 2.1 Plant material and growth conditions

The sweet potato varieties were cultivated at temperatures of between 22 and 28°C in the experimental base of the key laboratory of biology and genetic breeding for tuber and root crops in Chongqing, China. All *Arabidopsis thaliana* and *Nicotiana benthamiana* plants were grown in a 22°C and 28°C climate chamber (16 h light/8 h dark) in key laboratory of biology and genetic breeding for tuber and root crops in Chongqing, China.

### 2.2 Cloning *Ibβfruct2* family members and sequence analysis

Eight sweet potato cultivars—Yushu No. 2, Chaoshu No.1, S1-5, Suyu No. 1, Shangqiu 52-7, Yushu 33, Xinxiang, and Mianfen No.1—with various starch properties were used as sources for cloning *Ibβfruct2* family members. Total RNA was extracted from the leaf, stem, branch, and storage root of each cultivar using an RNAprep Pure Plant Kit (Tiangen Biotech, Beijing, China). A 5-mg, equally proportioned (w/w) mixture of the above RNAs was used as template for PCR amplification. Genomic DNA was extracted from fresh young leaves following the CTAB protocol (Kim and Hamada, 2005). Full-length genomic DNA and cDNA sequences of *Ibβfruct2* genes were cloned from the above-mentioned cultivars using primers FIbβfruct2 and RIbβfruct2. PCR products were recombined into the pEASY-T5 Zero Cloning vector (Transgen, Beijing, China) for sequencing. Primer sequences are listed in **Table S1**.

Sequences of *Ibβfruct2* members were subjected to BLASTn search against the sweet potato genome to obtain their pseudochromosome locations (Yang et al., 2017), and MapInspect 1.0 software (https://mapinspect.software.informer.com) was used to depict the physical locations. Multiple sequence alignment results from ClustalW were used for phylogenetic tree reconstruction with the neighbor-joining (NJ) method using MEGAX (Kumar et al., 2018). Tree reliability was measured using a bootstrap analysis with 1,000 replicates.

### 2.3 Detection of sequence variations in 507 sweet potato germplasms

To detect the sequence divergence of *Ibβfruct2* family members within the natural sweet potato population, genomic DNA of 507 previously reported sweet potato germplasms was amplified from an equivalently mixed DNA pool (Zhang et al., 2020a). As three *Ibβfruct2* family members (*Ibβfruct2-1*, *Ibβfruct2-2*, and *Ibβfruct2-3*) were found in sweet potato, three corresponding pairs of primers, FIbβfruct2-1A and RIbβfruct2-1A, FIbβfruct2-2A and RIbβfruct2-2A, and FIbβfruct2-3A and RIbβfruct2-3A, were designed for amplification of the three *Ibβfruct2* genes in the germplasms (**Table S1**). All amplicons were pooled and sequenced using the Illumina HiSeq 4000 platform, and all data analysis and variation calling were performed as previously described (Zhang et al., 2020a).

### 2.4 Gene expression pattern assay

Leaf, stem, branch, and storage root of Xushu 22 were sampled and diced at 95 days after transplanting and quickly frozen in liquid nitrogen then stored at −80°C until use for RNA extraction. Total RNA (1 μg) was extracted from each organ and reverse transcribed in a 20-μL volume using PrimeScript RT Master Mix (TaKaRa, Dalian, China). The expression pattern of three *Ibβfruct2* family members was detected using RT-qPCR as described previously (Zhang et al., 2017). Gene-specific primers were designed using sites specific to each gene and different from those in the other two family members. Primer pairs FIbβfruct2-1Q and RIbβfruct2-1Q, FIbβfruct2-2Q and RIbβfruct2-2Q, and FIbβfruct2-3Q and RIbβfruct2-3Q (**Table S1**) were used to detect the expression patterns of *Ibβfruct2-1*, *2*, and *3*, respectively. Fold changes were calculated according to the 2^–△△^ *^Ct^* method.

### 2.5 Cloning of *Ibβfruct2* promoters and *cis*-element prediction

The promoters of *Ibβfruct2* genes and their genomic sequences were amplified from genomic DNA of the eight sweet potato cultivars listed in section 2.1 by PCR using KOD FX Neo (TOYOBO) and the primers Pft2-2.5F and IbβfrTct2-mRNA-Rev (**Table S1**). The fragments obtained were ligated to the pEASY-T5 Zero Cloning vector (TransGen Biotech, Beijing, China) and sequenced. Sequences were analyzed using BLAST and Geneious Prime (Biomatters, Ltd., Auckland, New Zealand). *cis*-acting elements in promoters were predicted using PlantCARE (http://bioinformatics.psb.ugent.be/webtools/plantcare/html/; Lescot et al., 2002).

### 2.6 Assay of promoter activity under different treatments

The 2.5-kb promoter regions of the three *Ibβfruct2* family members were amplified from genomic DNA of sweet potato cultivars Xushu 22, Mianfen No. 1, and Xinxiang using primers pfrTct2.5-*Hind*III-Fwd and pfrTct2.5-*Nco*I-Rev (**Table S1**) and introduced into pCAMBIA1305.1 digested with *Hin*dIII and *Nco*I to generate promoter::GUS constructs. The recombinant constructs were transformed into *Agrobacterium tumefaciens* strain GV3101 and transiently expressed in *Nicotiana benthamiana* leaves. Infiltrated leaves were collected at 2 days post infiltration, and promoter activities in each sample were visualized using GUS staining, as described previously (Choi et al., 2020).

For low-temperature treatment, infiltrated *N. benthamiana* leaves were placed in a 4°C cold room for 2 days. For gibberellin (GA) treatment, leaves were sprayed with 50 μM GA (MERYER, Shanghai, China) and harvested for staining after 48 h. For light treatment, plants were grown in the dark for 24 h and then grown under light (800 lux) for 6 h.

### 2.7 Binding site prediction and yeast one-hybrid screening

Binding motifs of candidate transcription factor (TF) homologues and TF binding sites in the three *Ibβfruct2* promoters were predicted using PlantTFDB v5.0 (http://planttfdb.gao-lab.org). Promoter sequences (2.5 kb) were introduced into pAbAi to generate bait constructs, and positive colonies were screened from a sweet potato cDNA library previously generated (Zhang et al., 2020b) using the Matchmaker Gold Yeast One-Hybrid Library Screening System. Positive clones were sequenced and subjected to BLAST search against the NCBI database (https://www.ncbi.nlm.nih.gov).

### 2.8 Mutagenesis and yeast complementation assay

Conserved domains of the proteins encoded by the *Ibβfruct2* genes were analyzed using InterPro (http://www.ebi.ac.uk/interpro/) (Southan, 2000). *Ibβfruct2-1* mutants encoding proteins with deleted DUF3357, 32N, or 32C domains or deleted NDPNG or RDP motifs were obtained by PCR or overlap PCR using the pEASY-T5-*Ibβfruct2-1* construct as template and primers listed in **Table S1**.

Coding sequences (CDSs) of *Ibβfruct2-1* and *Ibβfruct2-1* with a 9-bp deletion (*Ibβfruct2-1M*) and the *Ibβfruct2-1* mutants were individually inserted into the yeast shuttle vector pDR196, containing URA3 as a selective marker. The new constructs were confirmed via sequencing and transformed into the invertase-deficient *Saccharomyces cerevisiae* strain SEY2102 using the PEG/LiAc method; transformants were selected on SD medium without uracil. The catalytic functions of proteins encoded by the *Ibβfruct2-1*, *Ibβfruct2-1M*, and *Ibβfruct2-1* mutants were determined by growth status of the transformed strains on SD medium plates and in 30 mL of SD liquid medium (-URA) for 3 days with sucrose as the sole carbon source. SEY2102 yeast cells transformed with the empty vector pDR196 were used as controls. Statistical significance was assessed using Student’s *t*-test. Probability values of less than 0.05 were considered significant, as indicated by asterisks in the figures.

### 2.9 Yeast complementation assay and invertase activity analysis

To confirm the function of proteins encoded by *Ibβfruct2* genes, the CDS of each *Ibβfruct2* family member, which showed highest identities with other CDS sequences of this gene member, or showed highest identities with the reference genomic sequence (Yang et al., 2017), were selected and inserted into the yeast shuttle vector pDR196 (Li et al., 2020) containing URA3 as a selective marker. The new plasmids were confirmed via sequencing. These plasmids and the empty vector pDR196 were transformed into the invertase-deficient *S. cerevisiae* strain SEY6210 (ATCC 96099) using the PEG/LiAc method, and transformants were selected on synthetic dropout (SD) medium without uracil. The catalytic function of proteins encoded by *Ibβfruct2-1*, *-2*, and *-3* was determined by growth status of transformed strains on SD medium plates and in 30 mL of SD liquid medium (-URA) for 3 days, with sucrose as the sole carbon source. SEY6210 yeast cells transformed with the empty vector pDR196 were used as a control.

To evaluate the invertase activities of proteins encoded by the three *Ibβfruct2* family members, yeast cells transformed with the three members were harvested by centrifugation at 5,000 rpm for 5 min, and crude enzyme extraction was performed using a previously reported method (Shang et al., 2018). Analysis of invertase activities was carried out using the DNS colorimetric method (Chang et al., 2017).

Statistical significance was assessed using Student’s *t*-test. Probability values of less than 0.05 were considered significant, as indicated by asterisks in the figures.

### 2.10 Molecular docking analysis

To understand the relationship between sequence variation and activity of proteins encoded by *Ibβfruct2* genes, their three-dimensional structures were predicted using I-TASSER (http://zhanglab.ccmb.med.umich.edu/I-TASSER); the structure of the sucrose molecule was downloaded from ZINC15 (http://zinc15.docking.org/). Docking of proteins and micromolecule sucrose was carried out using PyRx virtual screening software (https://pyrx.sourceforge.io/). Discovery Studio Visualizer (Dassault Systèmes BIOVIA, France) software was used to visualize and analyze the docked results.

### 2.11 Arabidopsis transformation and trait measurement

The full CDS of *Ibβfruct2-1* was recombined into the vector pEarleyGate101 using Gateway (Earley et al., 2006), yielding the *p35S*::*Ibβfruct2-1-YFP* construct. The construct was transformed into A. thaliana using the *Agrobacterium tumefaciens*–mediated floral dip method (Desfeux et al., 2000). Positive transgenic lines were identified by PCR detection of YFP using the primers YFP-Fwd and YFP-Rev and by detection of the *BAR* gene in the construct using the primers FBar and RBar. *Ibβfruct2-1* expression in the transgenic *A. thaliana* plants was detected using the RT-qPCR method described in the gene expression pattern assay section. The acid invertase activity was assayed as described previously (Lin et al., 2015). Thousand seed weight (g) was determined for 1000 seeds from each sample with three replicates. The starch and soluble sugar contents of leaves and seeds in transgenic and control *A. thaliana* plants were determined using a previously described method (Wu et al., 2021). The leaves and roots of 3-week-old seedlings were stained with an iodine solution (2% KI + 1% I2) and examined under a light microscope (Nikon, Japan), and images were captured using NIS-Elements BR 4.30.00 software as previously described (Wu et al., 2021).

## 3 Results

### 3.1 *Ibβfruct2* genes display sequence variations

To identify all the *Ibβfruct2* members in sweet potato accurately, we used a conserved primer pair to amplify *Ibβfruct2* in eight sweet potato varieties. After amplification and sequencing, 11 independent mRNAs and their corresponding genomic DNA sequences were obtained from these varieties. These sequences, combined with previously reported *Ibβfruct2* gene sequences, showed high levels of sequence variation in the intron regions but were conserved in the coding regions (**Figure S1**). They showed 84.32% to 99.98% genomic DNA sequence identity, 96.91% to 99.95% mRNA sequence identity, 96.56% to 99.95% CDS identity, and 97.26% to 99.85% identity of translated protein sequences. Among *Ibβfruct2* sequences obtained from different sweet potato varieties, the genomic DNA sequence identities ranged from 84.32% to 99.74%, and the CDS identity ranged from 96.56% to 99.75%. These results indicated high mRNA and protein sequence identities among the obtained *Ibβfruct2* gene sequences.

### 3.2 *Ibβfruct2* sequences represent three independent family members

**Figure 1:**
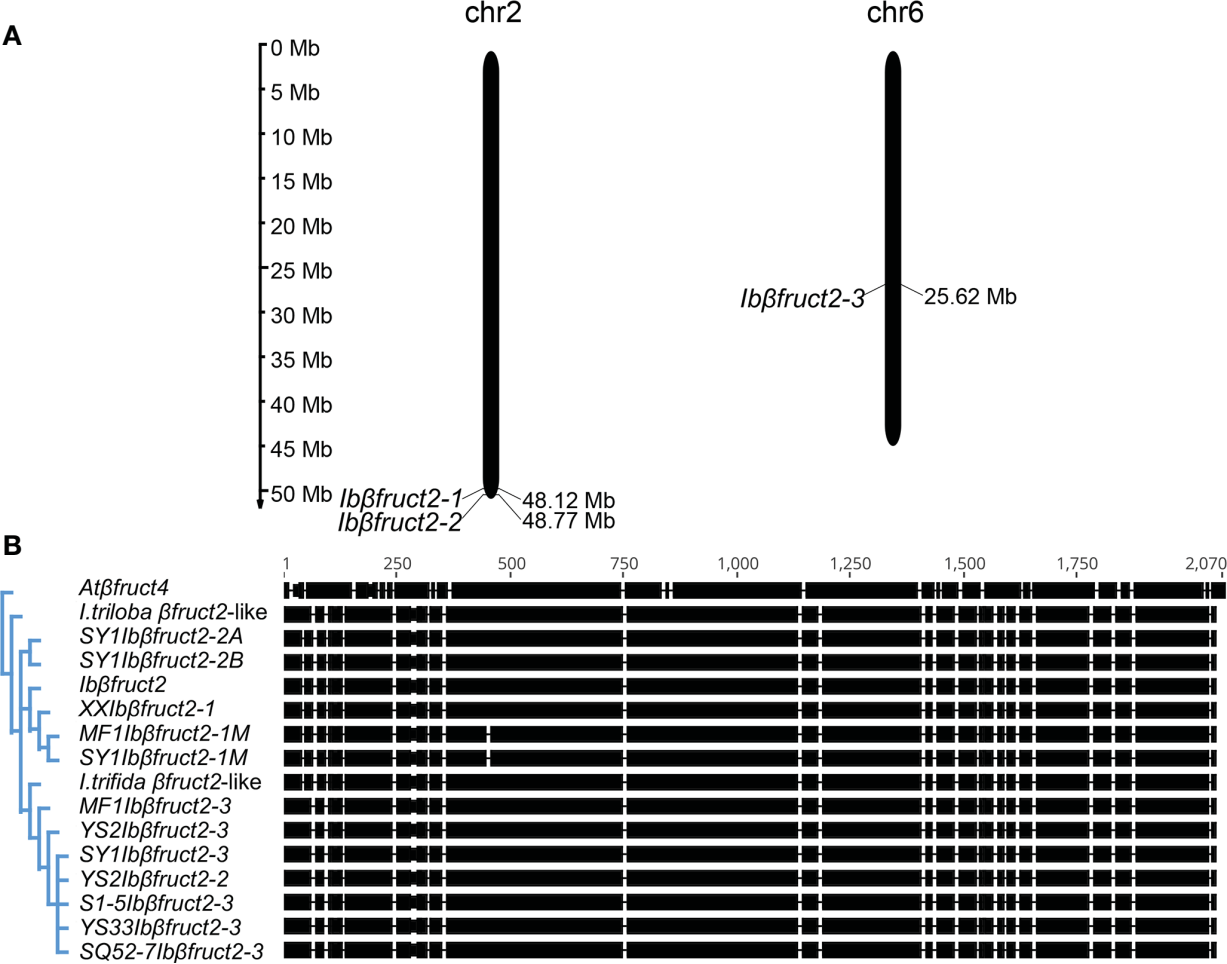
Sweet potato *Ibβfruct2* genes exhibit sequence differentiation. **(A)**. Location of *Ibβfruct2-1*, *Ibβfruct2-2*, and *Ibβfruct2-3* on sweet potato pseudochromosomes. The scale indicates the genome size of sweet potato (Mb). **(B)**. Phylogenetic analysis and CDS alignment of *Ibβfruct2* family members. *Atβfruct4*, *Arabidopsis thaliana* vacuolar invertase βFruct4 encoding gene At1G12240, AY142666.1; *I triloba βfruct2*-like, XM_031252273.1. *I trifida βfruct2*-like, part sequence of CP025646.1; S1-5, SY1, MF1, XX, YS2, YS33, and SQ52-7, the sweet potato varieties S1-5, Suyu No. 1, Mianfen No. 1, Xinxiang, Yushu No. 2, Yushu 33, and Shangqiu 52-7, respectively.

Although 11 independent *Ibβfruct2* sequences were cloned, they could only be mapped to three different loci on the 15 pseudochromosomes of the sweet potato genome, suggesting there are only three independent *Ibβfruct2* gene family members. *XXIbβfruct2*, which was cloned from sweet potato variety Xinxiang, shared 99.95% CDS identity with the previously reported *Ibβfruct2* gene (AY037937.1), but relatively lower (96.97% to 97.82%) CDS identity with other *Ibβfruct2* sequences (**Figure 1A**). This was regarded as *Ibβfruct2-1* members, located on pseudochromosome 2. Interestingly, an *Ibβfruct2-1* mRNA sequence with a 9-bp deletion (denoted *Ibβfruct2-1M*) was cloned from the cDNA of storage roots of Mianfen 1 and Suyu 1 (denoted as *MF1Ibβfruct2-1M* and *SY1Ibβfruct2-1M*, respectively). Two sequences cloned from Suyu No.1 shared high (99.95%) identity with each other, but relatively lower (97.17% to 97.82%) identity with other gene sequences (**Figure 1A**). These two genes were regarded as *Ibβfruct2-2* members, located at a separate locus close to *Ibβfruct2-1* on pseudochromosome 2. The remaining seven cloned *Ibβfruct2* sequences shared 98.28% to 99.75% CDS identity with each other, but relatively lower (96.97% to 97.72%) CDS identity with *Ibβfruct2-1* and *Ibβfruct2-2.* These sequences were mapped on pseudochromosome 6 and regarded as *Ibβfruct2-3* members (**Figure 1A**). The lengths of the *Ibβfruct2-1*, *Ibβfruct2-2*, and *Ibβfruct2-3* CDSs were 1,974, 1,974, and 1,977 bp, respectively.

To explore the evolutionary relationship among *Ibβfruct2* family members, we reconstructed a phylogenetic tree using all cloned *Ibβfruct2* sequences and their potential homologues from *Ipomoea* species. To obtain potential homologues, sequences of *Ibβfruct2* family members were used in a BLASTn search against the genomes of *Ipomoea trifida* and *Ipomoea triloba*, two close extant relatives of sweet potato (Gao et al., 2020). Only one *Ibβfruct2*-like gene was found in the genomes of *I. trifida* and *I. triloba*. The *I. trifida Ibβfruct2*-like gene shared a high level of CDS identity with all three *Ibβfruct2* family members: 97.568% to 97.619% CDS identity with *Ibβfruct2-1*, 98.227% to 98.278% CDS identity with *Ibβfruct2-2*, and 97.319% to 98.230% CDS identity with *Ibβfruct2-3*. However, it shared a higher level of genomic DNA sequence identity with *Ibβfruct2-3* (94.360% to 95.986%) than with *Ibβfruct2-1* (93.930%) or *Ibβfruct2-2* (85.543% to 85.567%). When compared with the *I. trifida Ibβfruct2*-like gene, the *I. triloba Ibβfruct2*-like gene shared lower sequence identities with the cloned *Ibβfruct2* sequences: 83.654% to 93.440% genomic DNA identity and 96.611% to 97.724% CDS identity.

Phylogenetic analysis showed that the cloned *Ibβfruct2* sequences could be divided into three groups, corresponding to three *Ibβfruct2* family members (**Figure 1B**). The *I. triloba Ibβfruct2*-like gene was not assigned to the same phylogenetic group as any of the *Ibβfruct2* gene family members, but *I. trifida Ibβfruct2*-like was assigned to the same phylogenetic group as *Ibβfruct2-3* family members. These results confirmed the presence of three independent *Ibβfruct2* gene family members.

### 3.3 The three *Ibβfruct2* members are not variety specific

To determine whether the three *Ibβfruct2* family members are variety specific, we amplified *Ibβfruct2* genes from mixed DNA of 507 sweet potato germplasms using PCR and performed Illumina sequencing to obtain all *Ibβfruct2* sequence variations. *Ibβfruct2-1*-specific variations occurred with a frequency of 29.712% to 33.555% among all sequences, while the frequency of *Ibβfruct2-3*-specific variations was 64.999% to 66.936%, indicating the *Ibβfruct2-1*- and *Ibβfruct2-3*-specifc variations account for approximately one-third and two-thirds of the total captured variations, respectively (**Table 1**). We also detected variations among *Ibβfruct2-3* family members at the same genomic position.

**Table 1:** *Ibβfruct2* gene sequence variations in 507 germplasms.

Position on genomic DNA (as shown in **Figure S1**)	Genotype	*Ibβfruct2-1/2/3* specific	Count	Coverage	Frequency (%)
56	C	*Ibβfruct2-1*	1,893	5,914	32.009
	G	*Ibβfruct2-2*, *Ibβfruct2-3*	4,014	5,914	67.873
60	G	*Ibβfruct2-1*, *Ibβfruct2-2*	1,978	5,917	33.429
	A	*Ibβfruct2-3*	3,935	5,917	66.503
67	T	*Ibβfruct2-1*	1,885	5,917	31.857
	G	*Ibβfruct2-2, Ibβfruct2-3*	4,011	5,917	67.788
85–87	---	*Ibβfruct2-1, Ibβfruct2-2*	1,981	5,931	33.401
	GCG	*Ibβfruct2-3*	3,940	5,931	66.431
96	C	*Ibβfruct2-1, Ibβfruct2-2*	1,992	5,941	33.530
	G	*Ibβfruct2-3*	3,943	5,941	66.369
207	G	*Ibβfruct2-1*	224	698	32.092
	C	*Ibβfruct2-2, Ibβfruct2-3*	473	698	67.765
238	A	*Ibβfruct2-1*	776	2,518	30.818
	G	*Ibβfruct2-2, Ibβfruct2-3*	1,728	2,518	68.626
312	G	*Ibβfruct2-1*	2,166	6,527	33.185
	C	*Ibβfruct2-2, Ibβfruct2-3*	4,352	6,527	66.677
327	A	*Ibβfruct2-1*	2,217	6,607	33.555
	G	*Ibβfruct2-2, Ibβfruct2-3*	4,387	6,607	66.399
330	T	*Ibβfruct2-1*	2,168	6,541	33.145
	C	*Ibβfruct2-2, Ibβfruct2-3*	4,368	6,541	66.779
356	G	*Ibβfruct2-1, Ibβfruct2-2*	1,744	5,323	32.763
	C	*Ibβfruct2-3*	3,563	5,323	66.936
399	C	*Ibβfruct2-1, Ibβfruct2-2*	1,217	3,477	35.001
	G	*Ibβfruct2-3*	2,260	3,477	64.999
424	T	*Ibβfruct2-1, -2, -3*	2,692	2,701	99.667
	A	*Ibβfruct2-3*	9	2,701	0.333
1,025–1,026 (Intron)	AA	*Ibβfruct2-1*	2,031	6,492	31.285
	TG	*Ibβfruct2-2, Ibβfruct2-3*	4,461	6,492	68.715
1,087–1,088	TG	*Ibβfruct2-1*	1,572	5,291	29.712
	AC	*Ibβfruct2-2, Ibβfruct2-3*	3,718	5,291	70.270
3,918	T	*Ibβfruct2-1, -2, -3*	2,520	3,396	74.205
	A	*Ibβfruct2-3*	869	3,396	25.589

*Ibβfruct2-2*-specific variations were not detected. At five sites, *Ibβfruct2-2* showed the same genotype as *Ibβfruct2-1* but a different genotype from *Ibβfruct2-3*; the frequency of these variations was 32.763% to 35.001%. At nine sites, *Ibβfruct2-2* showed the same genotype as *Ibβfruct2-3* but a different genotype from *Ibβfruct2-1*; the frequency of these variations was 66.399% to 70.270%. These results confirmed the presence of variations among *Ibβfruct2* gene sequences and indicated that the three *Ibβfruct2* members are not variety specific but exist together in each genome of most sweet potato genotypes.

### 3.4 The three *Ibβfruct2* members show differential expression patterns

**Figure 2:**
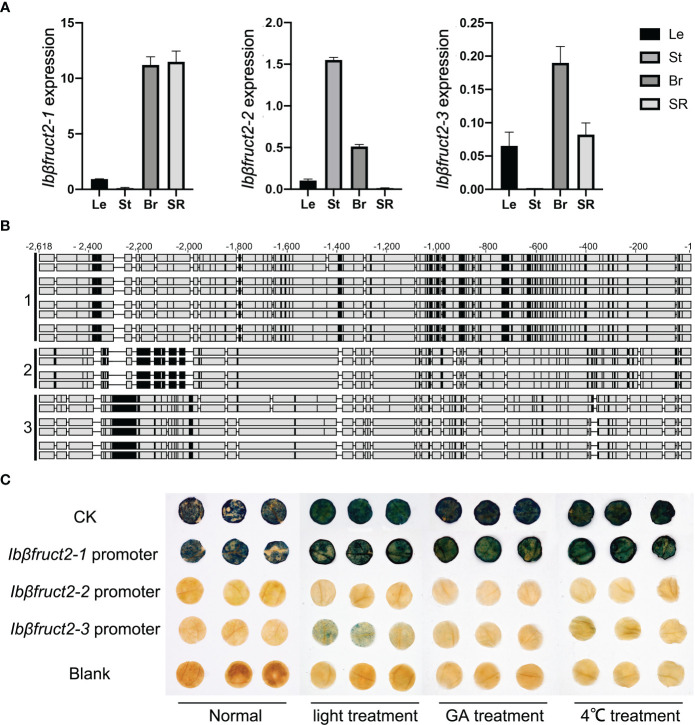
Three *Ibβfruct2* family members show divergence in expression patterns, promoter sequences, and activities. **(A)**. RT-qPCR detection of *Ibβfruct2-1*, *Ibβfruct2-2*, and *Ibβfruct2-3* expression patterns in leaf (Le), stem (St), branch (Br), and storage root (SR) of sweet potato. Error bar represent SEM calculated from three replicates. **(B)**. Alignment of 2.5-kb *Ibβfruct2* promoter sequences cloned from different sweet potato varieties displaying sequence differences among promoters of the three *Ibβfruct2* family members. 1, 2, and 3: promoter sequences of *Ibβfruct2-1*, *Ibβfruct2-2*, and *Ibβfruct2-3*, respectively. **(C)** Promoter-GUS fusion assays of promoter activities under normal conditions and light, GA, and low-temperature treatment.

Previous studies showed that the expression level of *Ibβfruct2* was negatively correlated with starch content of storage roots (Zhang et al., 2017). We further detected the expression of the *Ibβfruct2* family members in other tissues in sweet potato using RT-qPCR. *Ibβfruct2-1* had higher expression levels than the other two members of the family, and *Ibβfruct2-3* showed very low expression (**Figure 2A**). Furthermore, *Ibβfruct2-1* showed high expression in branches and storage roots and low expression in leaves and stems, while *Ibβfruct2-3* was highly expressed in branches but barely expressed in stems. *Ibβfruct2-2* showed high expression in branches and stems, but low expression in leaves and storage roots. These results indicate that the three *Ibβfruct2* family members have divergent expression patterns in different organs, suggesting their functional divergence in sweet potato.

### 3.5 *Ibβfruct2* gene members possess three different promoter sequences

To determine why and how the three *Ibβfruct2* members showed divergent expression patterns, we cloned the promoter sequences of the three *Ibβfruct2* family members from eight sweet potato varieties, finding that they differed in sequence (**Figure 2B**). We derived 24 promoter sequences from upstream of *Ibβfruct2-1*, which showed sequence identities of 97.683% to 99.951% between each other, 83.399 to 83.907% with *Ibβfruct2-2* promoter sequences, and 78.836% to 79.123% with *Ibβfruct2-3* promoter sequences. Nineteen promoter sequences of *Ibβfruct2-2* were obtained, showing 97.820% to 100% identity between each other and sharing 82.495% to 82.760% identity with *Ibβfruct2-3* promoter sequences. Three *Ibβfruct2-3* promoter sequences were obtained, which shared 99.957% sequence identity. These results thus revealed meaningful differences in promoter sequence among *Ibβfruct2-1*, *Ibβfruct2-2*, and *Ibβfruct2-3*.

### 3.6 The three *Ibβfruct2* promoters contain various *cis*-acting elements

To clarify whether the sequence variation in *Ibβfruct2* promoters is related to *cis*-acting elements and might thus affect gene expression patterns, we analyzed the three *Ibβfruct2* promoters to predict the presence of *cis*-acting elements using PlantCARE. The three promoters contained not only the common *cis*-acting elements CAAT-box and TATA-box, but also several other important elements such as the CGTCA-motif and TGACG-motif involved in response to methyl jasmonate, the wound-responsive element WUN-motif, the CAT-box related to meristem expression, the ABRE element involved in abscisic acid (ABA) responsiveness, and a number of *cis*-acting regulatory elements involved in light responsiveness, such as the G-Box, GT1-motif, MRE, and TCT-motif.

The three promoters contained characteristic elements. The AE-box, which is part of a module for light response, and the LTR element involved in low-temperature responsiveness were predicted in the *Ibβfruct2-1* promoter. The GA-responsive element GARE-motif, the GCN4_motif involved in endosperm expression, and TC-rich repeats involved in defense and stress responsiveness were predicted in the *Ibβfruct2-2* promoter, and the AT1-motif of the light-responsive module was predicted in the *Ibβfruct2-3* promoter (**Table S2**).

These results indicated that *Ibβfruct2* expression might be regulated by light and phytohormones and that *Ibβfruct2* is involved in plant growth and development and stress responses. Furthermore, the three *Ibβfruct2* promoters might be regulated by different factors and play different roles.

### 3.7 The three *Ibβfruct2* promoters possess different activities

To detect the activities of these three promoters, we selected a 2.5-kb *Ibβfruct2-1* promoter sequence obtained from Xushu 22, an *Ibβfruct2-2* promoter sequence obtained from Mianfen No. 1, and an *Ibβfruct2-3* promoter sequence obtained from Xinxiang, which was the most frequent sequence or showed highest identities with other sequences among the obtained promoter sequences of each *Ibβfruct2* member, and used them to construct three promoter::GUS vectors that were transiently transformed into *N. benthamiana* leaves. Transient expression of the promoter::GUS construct driven by the *Ibβfruct2-1* promoter yielded leaves that were stained dark blue (**Figure 2C**), indicating that the *Ibβfruct2-1* promoter could drive high levels of GUS activity, similar to the 35S promoter used in the positive control, and consistent with the high expression level of *Ibβfruct2-1* in sweet potato (**Figure 2A**). However, GUS activity driven by the *Ibβfruct2-2* and *Ibβfruct2-3* promoters was very low, indicative of low activity of these two promoters (**Figure 2C**).

We considered that the *Ibβfruct2-2* and *Ibβfruct2-3* promoters might be inducible. To assess this hypothesis, we applied low-temperature (4°C), GA, and light treatments to *N. benthamiana* leaves infiltrated with the promoter::GUS constructs, according to the characteristic *cis*-acting elements found in the three promoters. The *Ibβfruct2-1* promoter drove high levels of GUS activity in untreated and in low-temperature-, GA-, and light-treated *N. benthamiana* leaves, suggesting that it is a constitutive promoter (**Figure 2C**).

*N. benthamiana* leaves transiently expressing the *Ibβfruct2-3* promoter::GUS construct turned blue after GUS staining under light induction and exhibited blue dots under induction by GA and low temperature, indicating that the *Ibβfruct2-3* promoter is inducible. However, transient expression of the *Ibβfruct2-2* promoter::GUS construct in *N. benthamiana* produced no GUS staining under GA, low temperature, or light induction (**Figure 2C**), revealing that *Ibβfruct2-2* promoter activity was very low and could not be induced by these treatments. These results suggested that the promoters of the three *Ibβfruct2* family members differ in activities and expression characteristics.

### 3.8 The three promoters are specifically bound by different TFs

We next considered that the expression and promoter activities of the three *Ibβfruct2* members might regulated by different TFs. To test this hypothesis, and to explore the transcriptional regulation mechanism of the *Ibβfruct2* family members, we first predicted TF binding motifs in the three promoters using PlantTFDB v5.0. We detected 499, 522, and 486 potential binding sites in the promoters of *Ibβfruct2-1*, *Ibβfruct2-2*, and *Ibβfruct2-3*, respectively. All three promoters contained 28 types of common TF binding sites, including AP2, B3, BBR-BPC, BES1, bHLH, bZIP, and C2H2. However, each *Ibβfruct2* promoter also contained binding sites specific to that promoter. The *Ibβfruct2-1* promoter contained FAR1 (involved in light induction), LFY, and WOX (involved in developmental process). The *Ibβfruct2-2* promoter contained E2F/DP (involved in cell cycle and proliferate) and SRS (involved in GA response). The *Ibβfruct2-3* promoter contained an ARF binding site associated with auxin response elements (**Table S3**).

We further conducted a yeast one-hybrid assay to identify TFs binding to the three *Ibβfruct2* promoters. Using 1,000, 300, and 650 ng/mL Aureobasidin A (AbA) to suppress basal expression, respectively, we detected several potential TFs using the three promoters as bait. One TF ortholog of the *I. triloba* zinc finger protein ZAT10-like was found to potentially bind the *Ibβfruct2-1* promoter, while DUF724 domain–containing protein 6-like was detected using the *Ibβfruct2-2* promoter as bait. Seven potential TFs bound to the *Ibβfruct2-3* promoter: ethylene-responsive transcription factor 2 (ERF2), the homeobox-leucine zipper protein HAT4, the auxin-induced protein AUX22-like, the zinc finger protein LIGHT-DEPENDENT SHORT HYPOCOTYLS 1 (LSD1)-like, LSD10-like, stress-associated protein 5 containing the zinc finger A20 and AN1 domains, and stress-associated protein 8 containing the zinc finger A20 and AN1 domains. No common TFs were detected when using the three types of promoters individually as bait, indicating that the three promoters might be bound by different TFs and transcription of three *Ibβfruct2* family members might be regulated under different mechanisms.

### 3.9 The *Ibβfruct2-1* variant encodes a protein without invertase function

**Figure 3:**
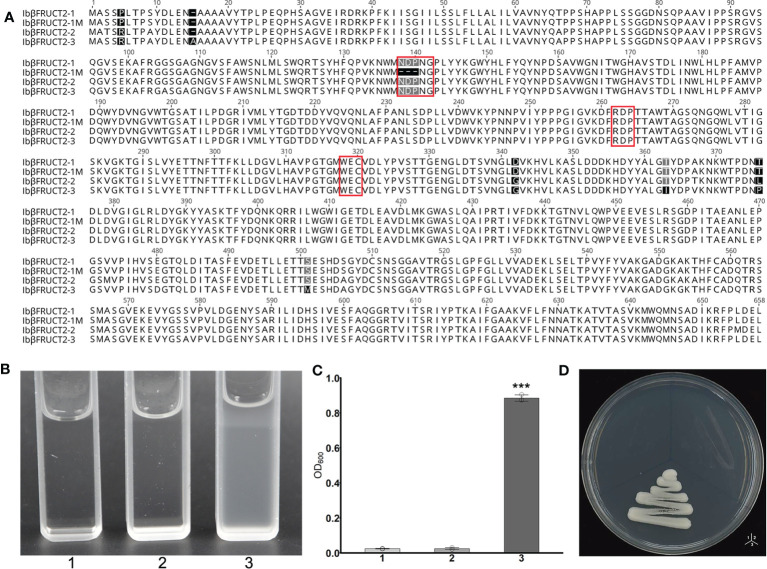
The *Ibβfruct2-1* variant encodes a protein deficient in invertase function. **(A)**. Comparison of amino acid sequences encoded by *Ibβfruct2* genes. Conserved motifs are boxed. **(B, C)**. Growth of invertase-deficient yeast strain SEY2102 transformed with 1. pDR196, 2. pDR196-Ibβfruct2-1, or 3. pDR196-Ibβfruct2-1M in liquid medium **(B)** and on solid sodium **(D)** containing sucrose as the sole carbon source. **(C)** OD_600_ of cultures in **(B)**. Error bars represent SEM calculated from at least three replicates. Asterisks indicate significant difference (***p < 0.001).

As the *Ibβfruct2* family members showed divergent expression patterns, we considered that they possessed functional divergence at the translation level. Interestingly, the 9-bp deletion in *Ibβfruct2-1M* mRNA when compared with that of *Ibβfruct2-1* would result in deletion of three amino acids in the deduced proteins, compared with the protein encoded by *Ibβfruct2-1* (denoted IbβFRUCT2-1, **Figure 3A**), and these three amino acids (NDP) were located in the highly conserved NDPNG motif, which is critical for invertase activity (Chen et al., 2009).

To check if this 3-aa deletion would affect the function or invertase activity of IbβFRUCT2 proteins, we inserted the *Ibβfruct2-1* and *Ibβfruct2-1M* CDSs into the yeast expression vector pDR196 to generate constructs pDR196-Ibβfruct2-1 and pDR196-Ibβfruct2-1M, respectively, and transformed each into the yeast triple mutant strain SEY2102, which lacks endogenous invertase activity and is unable to grow on a medium with sucrose as the sole carbon source. The results showed SEY2102 yeast cells transformed with pDR196-Ibβfruct2-1 could grow on medium containing sucrose as the sole carbon source (**Figures 3B, C, D**), indicating that IbβFRUCT2-1 exhibits invertase activity to hydrolyze sucrose to glucose as an available carbon source for SEY2102. By contrast, SEY2102 yeast cells transformed with empty pDR196 vector or transformed with pDR196-Ibβfruct2-1M were unable to grow on medium containing sucrose as the sole carbon source (**Figures 3B, C, D**), indicating that IbβFRUCT2-1M lacks invertase activity and that *Ibβfruct2-1M* might be a non-functional variant. These results also indicated that the NDP motif is critical for the function of the vacuolar invertase IbβFRUCT2.

### 3.10 Conserved domains and motifs are critical for invertase activity of IbβFRUCT2

**Figure 4:**
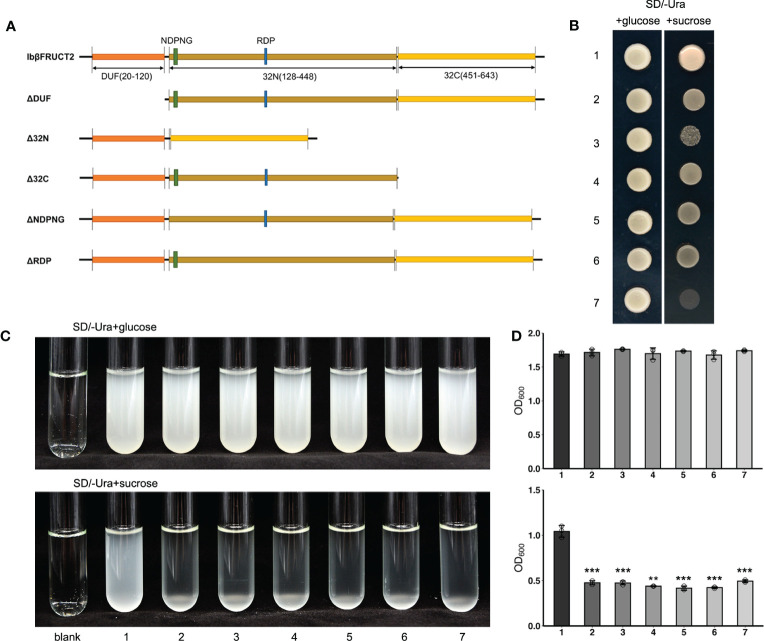
Yeast complementation tests identify domains and motifs critical for invertase function of IbβFRUCT2-1. **(A)**. Schematic representation of IbβFRUCT2 mutants. **(B, C)**. SEY2102/ura3 transformed with pDR196-*Ibβfruct2-1* (1), pDR196-ΔDUF (2), pDR196-Δ32N (3), pDR196-Δ32C (4), pDR196-ΔNDPNG (5), pDR196-ΔRDP (6) or pDR196 (7) and grown on solid medium **(B)** or in liquid medium **(C)** with glucose or sucrose as the sole carbon source. **(D)** OD_600_ of cultures in **(C)**. Error bars represent SEM calculated from at least three replicates. Asterisks indicate significant differences (**p < 0.01, ***p < 0.001).

We then further assessed the roles of active sites, conserved domains, and motifs for the enzyme activity of IbβFRUCT2-1. IbβFRUCT2 belongs to the glycoside hydrolases family 32 (GH32) and three conserved domains in the IbβFRUCT2 protein sequence were predicted by Uniprot: DUF3357 (DUF), the glycosyl hydrolase family 32 domain Glyco_hydro_32N (32N), and Glyco_hydro_32C (32C). We therefore generated additional *Ibβfruct2-1* mutants containing IbβFRUCT2 lacking the DUF domain (ΔDUF), the 32N domain (Δ32N), or the 32C domain (Δ32C) and examined these in SEY2102. Furthermore, we produced and tested mutants containing IbβFRUCT2 proteins lacking the NDPNG and RDP motifs (ΔNDPNG and ΔRDP, respectively, **Figure 4A**), which are critical for invertase activity. SEY2102 cells transformed with each of the IbβFRUCT2 mutants grew normally on solid or liquid medium with glucose as a carbon source, but did not grow on/in medium with sucrose as a carbon source, indicating that all the mutants lack invertase activity and cannot hydrolyze sucrose to glucose as an available carbon source for these cells (**Figures 4B–D**). Thus, all of the domains and motifs in IbβFRUCT2 are crucial for sucrose hydrolase function.

### 3.11 Proteins encoded by *Ibβfruct2-2* and *Ibβfruct2-3* have low invertase activities

**Figure 5:**
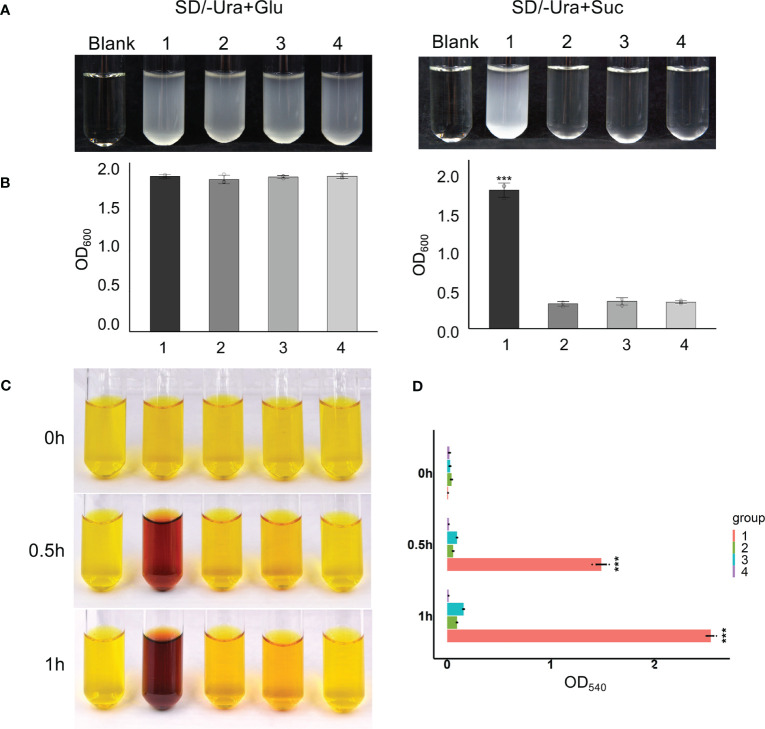
Functional identification of proteins encoded by *Ibβfruct2-1*, *Ibβfruct2-2*, and *Ibβfruct2-3* **(A)**. SEY6210 yeast cells transformed with pDR196-*Ibβfruct2-1* (1), pDR196-*Ibβfruct2-2* (2), pDR196-*Ibβfruct2-3* (3), or pDR196 (4) and grown in liquid sodium using glucose and sucrose as the sole carbon source. **(B)**. OD_600_ of cultures in **(A)**. **(C, D)**. Enzymatic activity analysis of IbβFRUCT2-1, IbβFRUCT2-2, and IbβFRUCT2-3 using DNS reaction. Color changes **(C)** and OD_540_ in colorimetric test **(D)** of the reaction buffer during reaction of a crude enzyme extract from yeast strain SEY6210 containing pDR196-*Ibβfruct2-1* (1), pDR196-*Ibβfruct2-2* (2), pDR196-*Ibβfruct2-3* (3), or pDR196 (4) with sucrose for 0, 0.5, or 1 h. Error bars represent SEM calculated from at least three replicates. Asterisks indicate significant difference (***p < 0.001).

The protein sequences of the three *Ibβfruct2* family members did not show differences in the domains or motifs tested (**Figure 3A**). We therefore examined if the proteins encoded by the three *Ibβfruct2* family members showed differences in protein function or enzyme activity. *Ibβfruct2-1*, *Ibβfruct2-2*, and *Ibβfruct2-3* CDSs were inserted separately into the pDR196 vector, and the vectors were transformed into the invertase-deficient yeast mutant strain SEY6210. SEY6210 yeast cells transformed with pDR196-*Ibβfruct2-1* could grow in medium containing sucrose as the sole carbon source, but those transformed with pDR196-*Ibβfruct2-2* or pDR196-*Ibβfruct2-3* could not (**Figures 5A, B**), indicating that their proteins (denoted IbβFRUCT2-2 and IbβFRUCT2-3, respectively) cannot hydrolyze sucrose to glucose to provide an available carbon source for SEY6210. Furthermore, when a crude enzyme extract of yeast strain SEY6210 containing pDR196-*Ibβfruct2-1* was incubated with sucrose for 0.5 and 1 h, DNS reaction buffer turned a deep brown color, confirming that IbβFRUCT2-1 has invertase activity and can hydrolyze sucrose to glucose and fructose (**Figure 5C**). Reaction of the crude enzyme extracts of yeast strain SEY6210 containing pDR196-*Ibβfruct2-2* or pDR196-*Ibβfruct2-3* with sucrose resulted in a lighter brown color in the DNS buffer when compared with the control, but the color intensity and OD value were significantly lower than those of pDR196-*Ibβfruct2-1* (**Figures 5C, D**), indicating that IbβFRUCT2-2 and IbβFRUCT2-3 possess very low invertase activities. These results showed that the protein products encoded by the three *Ibβfruct2* members differ in function and invertase activity, with only IbβFRUCT2-1 showing normal invertase function.

### 3.12 IbβFRUCT2 proteins differ in structure and affinity for the substrate

**Figure 6:**
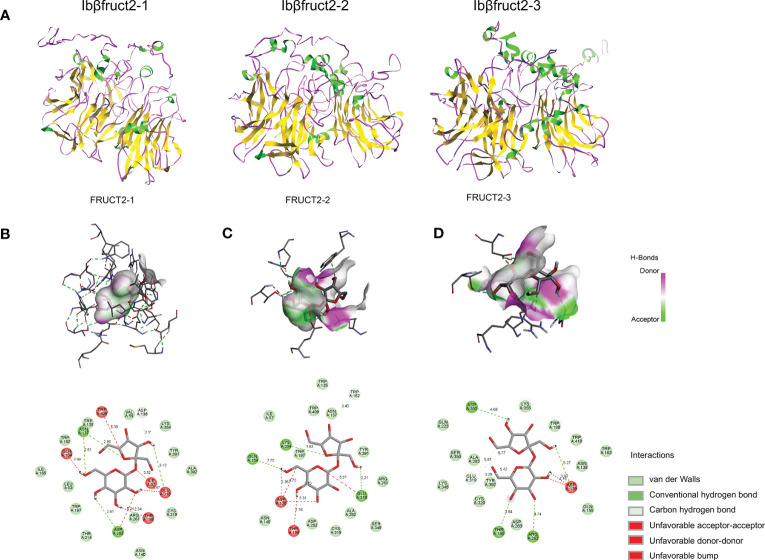
Prediction of the structures of three IbβFRUCT2 proteins and their interactions with sucrose. **(A)**. Three-dimensional structure of IbβFRUCT2-1, IbβFRUCT2-2, and IbβFRUCT2-3 predicted using I-TASSER. **(B-D)**. Docking of IbβFRUCT2-1 **(B)**, IbβFRUCT2-2 **(C)**, and IbβFRUCT2-3 **(D)** with sucrose and the amino acids participating in binding and their interactions with the sucrose molecule.

To assess why IbβFRUCT2-1 had normal invertase activity whereas IbβFRUCT2-2 and IbβFRUCT2-3 showed deficiencies in invertase function and very low enzymatic activity, we compared the amino acid sequences of the three proteins. We found only a few sequence variations: IbβFRUCT2-2 had two amino acid residues different from IbβFRUCT2-1, while IbβFRUCT2-3 had six amino acid residues different from IbβFRUCT2-1 (**Figure 3A**). Surprisingly, there were no sequence variations in the critical conserved domains or motifs, in the previously reported important amino acid residues within or around these motifs (Chen et al., 2009), or at the N-glycosylation sites (Tauzin et al., 2014). We then considered if the sequence variations among IbβFRUCT2 proteins might result in differentiation in protein structure and hence affect the distribution of the active sites or substrate binding sites. To test this, we predicted the theoretical structure of the three proteins using I-TASSER. Homology modeling results showed the three proteins exhibited different structural features. The IbβFRUCT2-1 protein structure contained 11 α-helixes and 32 β-sheets, the IbβFRUCT2-2 protein contained 12 α-helixes and 28 β-sheets, and the IbβFRUCT2-3 protein contained 15 α-helixes and 32 β-sheets (**Figure 6A**). Despite the differentiation in protein structures, docking results indicated the same active-site residues in the three proteins: Asp138/139 and Glu318/319 (Asp138 and Glu318 for IbβFRUCT2-1 and IbβFRUCT2-2, and Asp139 and Glu319 for IbβFRUCT2-3; **Table S4**).

To determine if differences in structure would affect binding affinity with the substrate, we performed molecular docking of each of the three IbβFRUCT2 proteins with a sucrose molecule. Van der Waals forces, hydrogen (H) bonds, and carbon-hydrogen (C-H) bonds were present between IbβFRUCT2-1 or IbβFRUCT2-2 proteins and sucrose molecules, as well as unfavorable bump, unfavorable acceptor-acceptor, and unfavorable donor-donor interactions (**Figures 6B, C**). Van der Waals forces, H-bonds, C-H bonds, and an unfavorable bump interaction were detected between IbβFRUCT2-3 protein and sucrose (**Figure 6D**).

When docking with IbβFRUCT2-1, the sucrose molecule was wrapped by the catalytic pocket formed by the active site residues, binding with Asn137 of the NDPNG motif, Glu 318 of the EC motif, and Asp262 of the RDP motif through H-bonds and with the active site Asp138 of NDPNG through a C-H bond. Furthermore, Cys319 of EC, Arg261 of RDP, and Trp135 around NDPNG were predicted to interact with sucrose via Van der Waals forces (**Figure 6B**).

In contrast, the sucrose molecule was not fully wrapped by the active site in IbβFRUCT2-2. When docking with sucrose, the active sites Glu318 and Asp138, and residues Gln154 and Lys354, bound sucrose through H-bonds (**Figure 6C**). However, unlike Asp138 of IbβFRUCT2-1, which binds to the fructose moiety of sucrose (**Figure 6B**), Asp138 of IbβFRUCT2-2 was predicted to bind the glucose moiety of sucrose (**Figure 6C**). IbβFRUCT2-3 had fewer amino acids participating in binding with sucrose. In IbβFRUCT2-3, Asp139 of NDPNG, Arg262 of RDP, and the residues Thr199 and Asp352 were predicted to bind the sucrose molecule via H-bonds; Cys320 of EC, Asn138 of NDPNG, and Asp263 of RDP participated in binding through Van der Waals forces. The active-site residue Glu318 was expected to bind sucrose through C-H bonds (**Figure 6D**). These results show that the amino acid residues and forces participating in binding with sucrose differed between IbβFRUCT2-1, IbβFRUCT2-2, and IbβFRUCT2-3.

To further assess their binding with sucrose, we calculated the binding affinities of the three IbβFRUCT2 proteins. As expected, IbβFRUCT2-1 showed the strongest binding affinity for sucrose (−6.5 kcal/mol) among the three proteins. The binding affinities of IbβFRUCT2-2 and IbβFRUCT2-3 were 2.5 and 0.5 kcal/mol, respectively, meaning they had very weak or little binding affinity for sucrose (**Table S4**). These results indicated that variations in several amino acid residues might cause changes in protein structures that further lead to changes in substrate binding and prevention of catalysis.

### 3.13 Heterologous expression of *Ibβfruct2-1* altered the starch and sugar content

**Figure 7:**
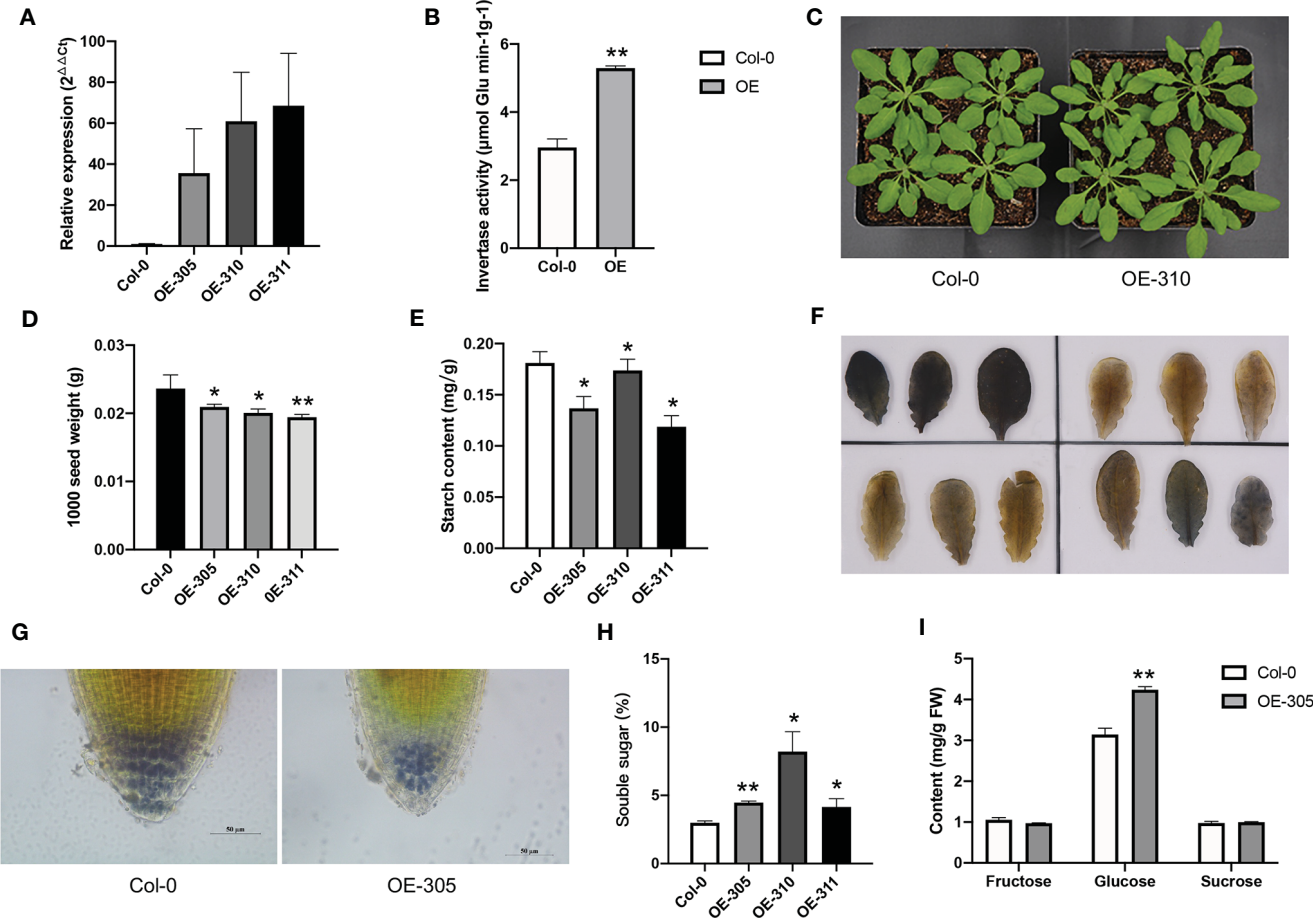
Heterologous expression of *Ibβfruct2-1* in Arabidopsis. **(A)**. Expression of *Ibβfruct2-1* in transgenic Arabidopsis plants detected using RT-qPCR. Col-0, wild type control; OE-305, OE-310 and OE-311 represent the three transgenic lines. **(B)**. Acid invertase activity in control and *Ibβfruct2-1* expressing plants. **(C)**. Phenotypes of control and *Ibβfruct2-1* expressing plants. **(D)**. 1000 seed weight of control and *Ibβfruct2-1* expressing plants. **(E)**. Starch content in leaves. **(F)**. Iodine-stained starch in leaves of control (Col-0, upper-left) and transgenic plants (upper-right, OE-305; lower-left, OE-310; lower-right, OE-311, respectively). **(G)**. Iodine-stained starch in the root tips of control and transgenic plant, respectively. **(H)**. Soluble content in leaves. **(I)**. Fructose, glucose and sucrose content in leaves of control and transgenic plants. Error bars represent SEM calculated from at least three replicates. Asterisks indicate significant differences (*p < 0.05, **p < 0.01).

Since among the three *Ibβfruct2* members, only *Ibβfruct2-1* showed normal expression and invertase activity, we further identify if it has *in vivo* function in plants. We transformed the *p35S*::*Ibβfruct2-1-YFP* construct into wild-type (Col-0) *A. thaliana*, and three independent homozygous transgenic lines, designated OE-305, OE-310 and OE-311, were selected from the T_2_ progeny and used for further detection. RT-qPCR results showed that the *Ibβfruct2-1* was expressed in these transgenic lines (**Figure 7A**). The *Ibβfruct2-1* expressing plants showed higher acid invertase activity when compared with the wild-type control (**Figure 7B**), indicating IbβFRUCT2-1 had invertase activity. There were no differences in growth and development between the transgenic progeny and the wild-type control (**Figure 7C**), but the 1000 seed weights of transgenic lines were significantly lower than that of control (**Figure 7D**). In contrast to their wild-type-like appearance, the starch content in the leaves of transgenic lines were significantly lower than that in the control lines (**Figure 7E**). Furthermore, iodine staining in the leaves (**Figure 7F**) and in the root tips (**Figure 7G**) also confirmed that the starch content in the transgenic lines were lower than that in control plants. Meanwhile, the soluble sugar content in the leaves of the transgenic lines was significantly higher than that of control (**Figure 7H**). Sugar composition analysis showed the leaves of transgenic plants accumulated more glucose when compared with that of control plants, but similar fructose and sucrose with that of control (**Figure 7I**). These results indicated *Ibβfruct2-1* gene have function *in vivo* and its expression altered soluble sugar and starch content in *A. thaliana*.

## 4 Discussion

We cloned 11 *Ibβfruct2* sequences displaying nucleotide variations from various sweet potato genotypes. Sequence comparison, genome mapping, and phylogenetic tree analysis revealed that these sequences represent three *Ibβfruct2* family members harbored at three separate loci. BLAST search against the *I. trifida* and *I. triloba* genomes identified only one *Ibβfruct2*-like gene in each. The *Ibβfruct2*-like gene detected in *I. trifida* showed a high level of sequence identity with the cloned *Ibβfruct2* genes and is probably an ortholog of *Ibβfruct2*. *I. trifida* has been proposed to be both the progenitor of sweet potato and a species with which it has introgressed (Muñoz-Rodríguez et al., 2018). Only one *Ibβfruct2*-like gene was detected in the *I. trifida* genome, indicating that the *Ibβfruct2* family members are probably duplicated genes generated from polyploidization. Although *Ibβfruct2-3* clustered together with its potential *I. trifida* ortholog in the phylogenetic tree, it is difficult to distinguish which family member was the progenitor and how the others arose during the evolutionary process because all the *Ibβfruct2* family members obtained showed a similar level of sequence identity with the potential *I. trifida* and *I. triloba* orthologs; this is similar to the results of a previous study showing that the *I. batatas* alleles were more closely related to each other than to likely orthologs from *I. trifida* and *I. triloba* (Muñoz-Rodríguez et al., 2018).

To explore if all sweet potato genotypes contain the three *Ibβfruct2* family members, we searched for these genes in 507 sweet potato germplasms. The high level of identity among the *Ibβfruct2* sequences made it difficult to distinguish them using common genotyping methods. Thus, we performed pooled-DNA sequencing to capture the gene variation in 507 sweet potato cultivars, landraces, and wild varieties collected worldwide (Zhang et al., 2020a). Among the three loci, *Ibβfruct2-3* sequences were present in the highest frequency in the genome, with abundant variations among *Ibβfruct2-3* sequences. It is possible that *Ibβfruct2-3* was generated during the evolution process under low selection pressure, with sequences free to accumulate further mutations and increasingly diverging from the sequence from which they were derived. *Ibβfruct2-1* accounted for about one-third of *Ibβfruct2* sequences, and little variation was detected among *Ibβfruct2-1* sequences, indicating that *Ibβfruct2-1* is relatively evolutionarily conserved. However, *Ibβfruct2-2*, which is located near *Ibβfruct2-1* on pseudochromosome 2 (**Figure 1A**), always showed the same haplotype as either *Ibβfruct2-1* or *Ibβfruct2-3* at the single-nucleotide variations and no *Ibβfruct2-2*-unique haplotype was detected, indicating that *Ibβfruct2-2* might be derived from exchange of *Ibβfruct2-1* and *Ibβfruct2-3*. However, to explain why and how the three *Ibβfruct2* family members arose, deep mining of the genome information and evolutionary history of hexaploid sweet potato is required.

The three *Ibβfruct2* family members showed different expression patterns, suggesting that these duplicated genes have undergone expression subfunctionalization or neofunctionalization (Liu et al., 2015). The three *Ibβfruct2* members share high sequence identities at the CDS level but less in their promoter regions, indicating that divergence of the promoter sequence following gene duplication might have led to their expression divergence (Qiao et al., 2018). First, the promoters of the three *Ibβfruct2* members showed different activities. Promoter-GUS activity analysis suggested that the *Ibβfruct2-1* promoter has high activity, while *Ibβfruct2-2* and *Ibβfruct2-3* promoters show inducible or little activity. This is in accord with the high expression level of *Ibβfruct2-1* and low expression of *Ibβfruct2-2* and *Ibβfruct2-3* in sweet potato plants. Second, the three promoters contained both common and different *cis*-acting elements, indicating that gain and loss of *cis*-regulatory elements contained in promoters occurred after gene duplication (Qiao et al., 2018). Divergence in promoter sequences indicates that they are probably induced by different stimuli and regulated by different TFs, implying different regulatory mechanisms for the three *Ibβfruct2* members, contributing to their different expression patterns.

The three *Ibβfruct2* members also showed functional divergence. IbβFRUCT2-1 exhibits the normal function of invertase, hydrolyzing sucrose to glucose and fructose. However, the 9-bp deletion variant *Ibβfruct2-1M* is also present in the sweet potato genome but encodes a nonfunctional invertase. Previous studies have shown that duplicated genes generated from WGD events might quickly be lost, silenced, or retained, acquire new functions, or become nonfunctional (Flagel and Wendel, 2009; Wang et al., 2013; Liu et al., 2015). We presume that this *Ibβfruct2-1* variant was probably generated through gene duplication and accumulated degenerative mutations to become nonfunctional (Flagel and Wendel, 2009). The biological effect of this nonfunctional gene and whether it is involved in regulating expression of the functional gene also need further investigation. In addition, the lack of function is associated with a 9-bp nucleotides deletion resulting in deletion of the NDP residues of the highly conserved NDPNG motif, indicating that the NDP residues are the most important residues of this motif and are crucial for IbβFRUCT2-1 protein function.

Invertase is an important enzyme in plants, and the amino acid residues crucial to its function and activity have been identified (Chen et al., 2009; Tauzin et al., 2014; Shen et al., 2019). The DUF3357 domain contains a signal peptide for vacuolar sorting and may play a role in protein folding, protein targeting, or the control of enzyme activity (Sturm, 1999). Our yeast complementation assay results confirmed that mutations in this domain, and other previously reported conserved domains and motifs of acid invertases, result in functional deficiency of IbβFRUCT2 in catalyzing sucrose hydrolysis.

Interestingly, when compared with the sequence of IbβFRUCT2-1, the sequences of IbβFRUCT2-2 and IbβFRUCT2-3 show no differences in or around the critical residues; however, variations in several amino acid residues whose roles have not previously been revealed result in their low invertase activity. To identify the factors conferring functional deficiency of IbβFRUCT2-2 and IbβFRUCT2-3, we analyzed their protein structures and interaction with substrate. As expected, the three IbβFRUCT2 proteins show large differences in their protein structures but share the same active-site residues, Asp of the NDPNG motif and Glu of the EC motif, similar to the active sites reported in another invertases (Shen et al., 2019). Molecule docking results showed that the two active-site residues of all three proteins participate in binding with sucrose, but with different binding patterns. During sucrose hydrolysis, the nucleophilic Asp residue in the NDPNG motif of invertase attacks C-2 of the fructose moiety, forming a fructose-enzyme intermediate and releasing glucose (Chen et al., 2009). In IbβFRUCT2-2, binding of Asp138 to the glucose moiety but not the fructose moiety probably alters the formation of the fructose-enzyme intermediate, thus resulting in the prevention of catalysis. In IbβFRUCT2-1, previously identified critical residues in other invertases participate in the binding of protein with sucrose, and sucrose is wrapped completely by the catalytic pocket (**Figures 6A, B**). In IbβFRUCT2-3, these critical residues also participate in binding with sucrose, but with a different binding pattern than that of IbβFRUCT2-1. Fewer amino acid residues (and some different from those in IbβFRUCT2-1) participate in the binding of sucrose by IbβFRUCT2-3, indicating that incorrect binding sites and binding patterns might lead to weak sucrose binding affinity and functional deficiency of IbβFRUCT2-3. Differences in amino acid sequences result in differentiation of protein structures among the IbβFRUCT2 invertases, which might affect their binding patterns and affinities with the substrate, leading to functional differentiation. These results show that in addition to variation in critical active or conserved sites, variations in non-critical sites might also affect the function and activity of proteins.

Together, our results suggest that during the formation and evolution of hexaploid sweet potato, the *Ibβfruct2* progenitor(s) originated from a diploid and/or tetraploid progenitor. Duplicated genes were generated through polyploidization followed by loss, retention, silencing, subfunctionalization, or neofunctionalization. Mutations accumulating in gene sequences and promoter regions generated the present *Ibβfruct2* family members, which show sequence, regulatory, and functional divergence.

Duplicated genes can result from tandem duplication, from transposition to new chromosomes, or from WGD (Flagel and Wendel, 2009; Birchler and Yang, 2022). In sweet potato, duplicated genes might have evolved from the hybridization and WGD events and frequently exhibit accumulation of mutants and changes in gene expression or function under natural or artificial selection, to improve adaptive genetic diversity or lead to evolution of new traits and thus increased adaptability to new environmental conditions (Roulin et al., 2013; Yan et al., 2022).

Since *Ibβfruct2-2* and *Ibβfruct2-3* showed very low promoter activities, protein function, deficiency, and little enzymatic activity, why were these genes generated or preserved during the evolutionary process? Genes exert their biological roles in many different ways. It is possible that *Ibβfruct2-2* and *Ibβfruct2-3* are nonfunctional and might be lost during evolution, but maintain the duplicate state due to some forces (Birchler and Yang, 2022), or they may play specific roles in sweet potato. First, although the proteins encoded by *Ibβfruct2-2* and *Ibβfruct2-3* cannot complement the function of invertase in yeast, they do exhibit a low level of invertase activity in *in vitro* catalytic assays, indicating that they may have a function. Second, *Ibβfruct2-2* and *Ibβfruct2-3* show different tissue-specific expression patterns from *Ibβfruct2-1*, and the promoters of *Ibβfruct2-2* and *Ibβfruct2-3* contain various *cis*-acting elements involved in responses to environmental stimuli, hormones, developmental stages, and stress. It is possible that the expression of *Ibβfruct2-2* and *Ibβfruct2-3* could be regulated under specific conditions; thus, these genes are involved in different physiological processes. Furthermore, since *Ibβfruct2-2* is located near *Ibβfruct2-1* on pseudochromosome 2, whether the existence of *Ibβfruct2-2* affects the expression pattern of *Ibβfruct2-1* needs further investigation (Flagel and Wendel, 2009; Hahn, 2009). Our results indicate that the accumulation of mutants during the evolution of the hexaploid sweet potato genome might have occurred in both coding and promoter regions, altering gene structure, protein function, and regulation of expression in duplicated genes and implying that the origin and genome evolution of hexaploid sweet potato genome are very complicated.

The presence of several sequences displaying variations at each *Ibβfruct2* locus might result from the high level of heterozygosity of the sweet potato genome. On the basis of its origin and evolutionary process, multiple gene copies are assumed to be present in the hexaploid sweet potato genome; our recent study also revealed 1–37 potential homologs or paralogs of each gene in the sweet potato genome database studied (Zhang et al., 2020a). This causes additional problems for genetic modification in sweet potato. Although we obtained 11 independent *Ibβfruct2* sequences, only three *Ibβfruct2* loci were detected, and only one gene family member, *Ibβfruct2-1*, showed high transcription levels, high promoter activity, and normal invertase activity. Furthermore, this gene family member also had a nonfunctional variant (*Ibβfruct2-1M*). Our results indicate that many duplicated genes might have been generated in the sweet potato genome, but they might show differentiation in expression or function. Thus, for genetic modification of sweet potato, it is necessary to test the expression and protein functions of all gene family members to determine the strategies of gene manipulation.

It was reported that the reduced expression of VIN decreased the seed weight in rice and Arabidopsis (Xu et al., 2019; Vu et al., 2020), but our results showed heterologous expression of *Ibβfruct2-1* reduced the 1000 seed weight in Arabidopsis. There were two possible reasons. Firstly, IbβFRUCT2-1 showed high invertase activity in transgenic plants, and cleaved more sucrose than that in control plants, and promoted more photosynthetic product transport into vacuoles of leaves and roots, then reduced the transport and accumulation of carbohydrate in seeds; Secondly, as a key enzyme involved in sugar metabolism and signaling, the expression and activity of VIN was preciously regulated (Ruan et al., 2010). IbβFRUCT2-1 might interact with other components or signaling pathways to reduce the matter accumulation in seeds to maintain the homeostasis of carbohydrate metabolism and development of the whole plant. However, as a VIN encoding gene in sweet potato, which major sink organ was storage root, whether *Ibβfruct2-1* showed different roles with rice or Arabidopsis VIN genes in seed development process need to be further explored.

Heterologous expression of *Ibβfruct2-1* gene in *A. thaliana* altered starch and soluble sugar content in plants, indicating *Ibβfruct2-1* might be involved in starch and sugar metabolism in plants. *Ibβfruct2-1* expression decreased starch content in *A. thaliana* leaves and root tips, but increased sugar content, which consist with our previous supposition that it’s a negative regulator in starch content (Zhang et al., 2017). Based on the function of invertase that irreversibly hydrolyzing sucrose to fructose and glucose, the sucrose content was supposed to be decreased, and the fructose and glucose contents were supposed to be increased in the *Ibβfruct2-1* expressing lines. However, there was significant increase in glucose content, but no significant decrease in sucrose and increase in fructose content was detected in the transgenic plants, indicating glucose was the mainly increased soluble sugar. That might because the vacuolar invertase only catalyze the hydrolyzing of sucrose in vacuole, but the overall metabolism in the whole plants was a complex process and was dynamically regulated (Zeeman et al., 2010), thus the expression of *Ibβfruct2-1* didn’t affect the sucrose content in the whole plants. Our results confirmed the role of *Ibβfruct2-1* in starch and sugar metabolism, which reduced starch content and increased the content of soluble sugar, especially glucose content, and indicating its application potential in starch and sugar-related traits improvement in crops.

Furthermore, invertase activity can also be regulated at a post-translational level by proteinaceous inhibitors (Tauzin et al., 2014). Further investigation of the post-translational regulatory mechanism of IbβFRUCT2 is also essential to understand its regulatory mechanism. We systemically revealed detailed information on the sequence, regulatory, and functional divergence among members of a key enzyme-encoding gene family in allohexaploid sweet potato. These findings represent an important advance in understanding genome components and the regulatory and functional divergence among duplicated genes in sweet potato. They also provide useful information for functional studies of key genes, especially those involved in regulation of important traits, which are crucial for quality improvement through genetic engineering and genome editing–assisted breeding in sweet potato and other polyploid crops.

## 5 Conclusion

Even though multiple *Ibβfruct2* gene family members exist in the sweet potato genome, only *Ibβfruct2-1* showed a high expression level, promoter activity, and normal protein function. Furthermore, variations in protein sequences can lead to functional deficiency even without affecting key residues. Our results indicate that the evolution and regulation of duplicated genes in sweet potato are precise and complex; thus, the genetic improvement of this species will require a clear understanding of the functional divergence among gene family members.

## Data availability statement

The original contributions presented in the study are included in the article/**Supplementary Material**. Further inquiries can be directed to the corresponding authors.

## Author contributions

KZ, XW and ZW conceived and designed the experiments. XJ, CY and HH performed the experiments. YF and CY analyzed the data. DT, CL, QC and JW contributed reagents/materials/analysis tools. KZ wrote the paper. KZ interpreted data and edited the manuscript. All authors contributed to the article and approved the submitted version.

## Supplementary material

Supplementary Figure 1 Alignment of genomic DNA sequences of cloned *Ibβfruct2* genes displaying sequence differentiation in intron regions. The 1^st^, 2^nd^, 3^rd^, 4^th^, and 5^th^ intron sequences are marked with red, green, orange, blue, and purple lines, respectively. Start and stop codons are marked as dark lines. SY1, MF1, XX, YS2, S1-5, YS33, and SQ52-7, sweet potato varieties Suyu No. 1, Mianfen No. 1, Xinxiang, Yushu No. 2, S1-5, Yushu 33, and Shangqiu 52-7, respectively.
