# Supplementary material for: Regulatory and functional divergence among members of Ibβfruct2, a sweet potato vacuolar invertase gene controlling starch and glucose content
Source: Front Plant Sci. 2023 Jun 27;14:1192417. doi: 10.3389/fpls.2023.1192417 (PMC10333694; doi:10.3389/fpls.2023.1192417)
Supplement: Supplementary file 2 [file Table_1.docx]

Table S1 Primers used in this study.

| **Primers** | **Sequences** | **Use** |
| --- | --- | --- |
| FIbβfruct2 | 5’-CATATGTCAGTCATTCATAGTTGACAAC -3’ | *Ibβfruct2* genes cloning |
| RIbβfruct2 | 5’-ATACTTGTAAACTCATCATTTTGATTCAAA-3’ |  |
| FIbβfruct2-1A | 5’-AACTTTGTCGGAAACCCATGGCTAG-3’ | Gene variations detection |
| RIbβfruct2-1A | 5’-CCCAAACAGCGGAATCTGGATTGT-3’ |  |
| FIbβfruct2-2A | 5’-CCATTATTCTTGGACAGGGCTCACC-3’ |  |
| RIbβfruct2-2A | 5’-TTCCACTTCTTCAACCGGCCATTG-3’ |  |
| FIbβfruct2-3A | 5’-GGATGGATTGGTGAAACTGATTTAGAAGC-3’ |  |
| RIbβfruct2-3A | 5’-CCCCCATATAATCACTTCATATCTCG-3’ |  |
| DUF-F | GGGCTGCAGGAATTCAGTTGGCAACGAACGAGTTA | Construction of mutant expressing ΔDUF |
| SIFRUCT2-R | CTTGATATCGAATTCCTACAACTCATCCAAGGGGA |  |
| SIFRUCT2-F | GGGCTGCAGGAATTCATGGCTAGCTCTCCTTTGAC | Construction of mutant expressing Δ32N |
| SIFRU-32NR | AAGCTTTCCACTTCTTCAACGTAACTCGTTCGTTGCCAAC |  |
| SFRU-32NF | GTTGGCAACGAACGAGTTACGTTGAAGAAGTGGAAAGCTT |  |
| SIFRUCT2-R | CTTGATATCGAATTCCTACAACTCATCCAAGGGGA |  |
| SIFRUCT2-F | GGGCTGCAGGAATTCATGGCTAGCTCTCCTTTGAC | Construction of mutant expressing Δ32C |
| SIFRU-32CR | CTTGATATCGAATTCTTCAACCGGCCATTGAAGCA |  |
| SIFRUCT2-F | GGGCTGCAGGAATTCATGGCTAGCTCTCCTTTGAC | Construction of mutant expressing ΔNDPNG |
| NDPNG-F | CCTGTGAAGAATTGGATGCCATTGTACTACAAAGGATGG |  |
| NDPNG-R | CCATCCTTTGTAGTACAATGGCATCCAATTCTTCACAGG |  |
| SIFRUCT2-R | CTTGATATCGAATTCCTACAACTCATCCAAGGGGA |  |
| SIFRUCT2-F | GGGCTGCAGGAATTCATGGCTAGCTCTCCTTTGAC | Construction of mutant expressing ΔRDP |
| RDP-R | CCACGCAGTGGTAAAGTCCTTGAC |  |
| RDP-F | GTCAAGGACTTTACCACTGCGTGG |  |
| SIFRUCT2-R | CTTGATATCGAATTCCTACAACTCATCCAAGGGGA |  |
| FIbβfruct2-1Q | 5’-GGTCTCGACCACAGGAGAAAACGGAC-3’ | *Ibβfruct2-1* expression detection |
| RIbβfruct2-1Q | 5’- TCCAATACCCACATCCAAATCTGTG-3’ |  |
| FIbβfruct2-2Q | 5’-GCACTTGGAACATATGATCCCACG-3’ | *Ibβfruct2-2* expression detection |
| RIbβfruct2-2Q | 5’-TTGAAGCACGTTTGTCCCAGTCTTC-3’ |  |
| FIbβfruct2-3Q | 5’- GGTTTCGACCACCGGAGAAAACGGGC-3’ | *Ibβfruct2-3* expression detection |
| RIbβfruct2-3Q | 5’- CCCGATACCCACATCCAAATCTGGG-3’ |  |
| Pft2-2.5F | 5’- TTGTTGGATTCACAATAATGCAAGAAATGTCG-3’ | Promoter sequences cloning |
| IbβfrTct2-mRNA-Rev | 5’-ATACTTGTAAACTCATCATTTTGATTC-3’ |  |
| pfrTct2.5-*HindI*II-Fwd | 5’-CAAGCTTTTGTTGGATTCACAATAATGCAAG-3’ | Promoter::GUS constructs |
| pfrTct2.5-*Nco*I-Rev | 5’-CCCATGGGGGTTTCCGACAAAGTTGTCAAC-3’ |  |
| YFP-Fwd | 5′-TGG TCGAGCTGGACGGCGACGTAAAC-3′ | Positive transgenic plants detection |
| YFP- Rev | 5′-TTCTCGTTGGGGTCTTTGCTCAGGGC-3′ |  |
| FBar | 5′-TGGGCAGCCCGATGACAGCGACCAC-3′ |  |
| RBar | 5′-ACCGAGCCGCAG GAACCGCAGGAGT-3′ |  |

Table S2 Cis-acting elements detected in the 3 types of *Ibβfruct2* promoters.

| Cis-acting elements | P1 | P2 | P3 | Sequence | Function |
| --- | --- | --- | --- | --- | --- |
| AAGAA-motif | Y | Y | Y | GAAAGAA |  |
| ABRE | Y | Y | Y | ACGTG | involved in the abscisic acid responsiveness |
| ABRE3a | Y | Y | Y | TACGTG |  |
| ABRE4 | Y | Y | Y | CACGTA |  |
| AT~ABRE | Y | Y | Y | TACGTGTC |  |
| AT~TATA-box | Y | Y | Y | TATATA |  |
| Box 4 | Y | Y | Y | ATTAAT | part of a conserved DNA module involved in light responsiveness |
| Box Ⅱ | Y | Y | Y | ACACGTAGA | part of a light responsive element |
| C-box | Y | Y | Y | ACGAGCACCGCC | involved in light responsiveness |
| CAAT-box | Y | Y | Y | CAAT | common cis-acting element in promoter and enhancer regions |
| CAT-box | Y | Y | Y | GCCACT | related to meristem expression |
| G-Box | Y | Y | Y | CACGTG | involved in light responsiveness |
| G-box | Y | Y | Y | CACGTG | involved in light responsiveness |
| GT1-motif | Y | Y | Y | GGTTAA | light responsive element |
| MRE | Y | Y | Y | AACCTAA | MYB binding site involved in light responsiveness |
| MYB | Y | Y | Y | CAACCA |  |
| MYC | Y | Y | Y | CATGTG |  |
| Myb | Y | Y | Y | TAACTG |  |
| TATA-box | Y | Y | Y | ATTATA | core promoter element around -30 of transcription start |
| TCT-motif | Y | Y | Y | TCTTAC | part of a light responsive element |
| W box | Y | Y | Y | TTGACC |  |
| WRE3 | Y | Y | Y | CCACCT |  |
| WUN-motif | Y | Y | Y | AAATTTCCT | wound-responsive element |
| chs-CMA2a | Y | Y | Y | TCACTTGA | part of a light responsive element |
| ACE | N | Y | Y | GACACGTATG | involved in light responsiveness |
| AuxRE | N | Y | Y | TGTCTCAATAAG | part of an auxin-responsive element |
| MYB-like | N | Y | Y | TAACCA |  |
| Myb-binding | N | Y | Y | CAACAG |  |
| CGTCA-motif | Y | N | Y | CGTCA | involved in the MeJA-responsiveness |
| STRE | Y | N | Y | AGGGG |  |
| TGACG-motif | Y | N | Y | TGACG | involved in the MeJA-responsiveness |
| as-1 | Y | N | Y | TGACG |  |
| TCA | Y | Y | N | TCATCTTCAT |  |
| AT1-motif | N | N | Y | AATTATTTTTTATT | part of a light responsive module |
| GARE-motif | N | Y | N | TCTGTTG | gibberellin-responsive element |
| GCN4_motif | N | Y | N | TGAGTCA | involved in endosperm expression |
| TC-rich repeats | N | Y | N | GTTTTCTTAC | involved in defense and stress responsiveness |
| AE-box | Y | N | N | AGAAACAA | part of a module for light response |
| Box Ⅲ | Y | N | N | ATCATTTTCACT | protein binding site |
| Myc | Y | N | N | TCTCTTA |  |
| LTR | Y | N | N | CCGAAA | involved in low-temperature responsiveness |

Table S3 Analysis of binding sites in promoters.

| Binding site | P1 | P2 | P3 |
| --- | --- | --- | --- |
| AP2 | 9 | 16 | 7 |
| B3 | 6 | 18 | 5 |
| BBR-BPC | 3 | 3 | 1 |
| BES1 | 1 | 2 | 6 |
| bHLH | 25 | 50 | 43 |
| bZIP | 64 | 84 | 68 |
| C2H2 | 25 | 14 | 22 |
| CPP | 3 | 2 | 2 |
| Dof | 29 | 49 | 31 |
| EIL | 2 | 2 | 3 |
| ERF | 61 | 54 | 55 |
| G2-like | 13 | 7 | 9 |
| GATA | 25 | 19 | 18 |
| GRAS | 3 | 2 | 2 |
| GRF | 1 | 1 | 1 |
| HD-ZIP | 35 | 47 | 28 |
| HSF | 20 | 18 | 18 |
| MIKC_MADS | 9 | 11 | 6 |
| MYB | 85 | 43 | 95 |
| MYB_related | 6 | 5 | 7 |
| NAC | 7 | 22 | 13 |
| NF-YB | 3 | 2 | 2 |
| RAV | 3 | 5 | 5 |
| S1Fa-like | 2 | 2 | 2 |
| TCP | 17 | 14 | 11 |
| Trihelix | 7 | 2 | 8 |
| WRKY | 24 | 5 | 13 |
| ZF-HD | 4 | 15 | 3 |
| FAR1 | 1 |  |  |
| LFY | 2 |  |  |
| WOX | 3 |  |  |
| SBP | 1 | 1 |  |
| E2F/DP |  | 5 |  |
| SRS |  | 1 |  |
| Nin-like |  | 1 | 1 |
| ARF |  |  | 1 |

Table S4 Enzyme Commission (EC) numbers, active sites and the binding affinity of three proteins with sucrose.

| **Protein** | **PDB Hit** | **CscoreEC** | **TM-score** | **RMSDa** | **IDENa** | **Cov** | **Active site residues** | **Binding affinity score** |
| --- | --- | --- | --- | --- | --- | --- | --- | --- |
| IbβfFRUCT2-1 | 1st8A | 0.502 | 0.792 | 1.15 | 0.466 | 0.804 | 138,318 | -6.5 |
| IbβFRUCT2-2 | 2ac1A | 0.529 | 0.785 | 1.46 | 0.466 | 0.804 | 138,318 | 2.5 |
| IbβFRUCT2-3 | 3ugfA | 0.580 | 0.785 | 1.41 | 0.466 | 0.802 | 139,319 | 0.5 |

(a)CscoreEC is the confidence score for the EC number prediction. CscoreEC values range in between [0-1]; where a higher score indicates a more reliable EC number prediction.

(b) TM-score is a measure of global structural similarity between query and template protein.

(c) RMSDa is the RMSD between residues that are structurally aligned by TM-align.

(d) IDENa is the percentage sequence identity in the structurally aligned region.

(e) Cov represents the coverage of global structural alignment and is equal to the number of structurally aligned residues divided by length of the query protein

(f) A lower score of binding affinity value indicates a stronger binding affinity. affinity > -4 kcal/mol，weak or little binding; -7 kcal/mol < Affinity <= -4 kcal/mol, moderate binding；Affinity <= -7 kcal/mol, strong binding.
